# Mania a Potu

**Published:** 1860-07

**Authors:** Warren Stone

**Affiliations:** Professor of Surgery in the University of Louisiana


### MANIA A POTU.

BY WARREN STONE, M. D., PROFESSOR OF SURGERY IN THE UNIVERSITY OF LOUISIANA.

This term is often applied to all the forms of delirium that are caused by the excessive use of stimulants, but it more properly belongs to the acute form, where the violence of the delirium, or mania, is mainly due to alcoholic blood-poison. The term delirium tremens is more appropriate to the more chronic form, where the delirium and tremor are due to the sudden privation of a long-accustomed stimulant. It is the former that I think is generally improperly treated, and is so often the cause of sudden and premature death. Brain fever and apoplexy are terms often kindly substituted, as being more respectable; but names do not alter facts. Mania a potu usually occurs with the robust who habitually use alcoholic stimulants, but not to any great excess, except upon occasions, and when they are carried to a certain extent, a necessity for their continuance is created, and their excessive use cannot, or will not, be resisted until the stomach gives way and finally rejects them. During this process the mucous membrane becomes engorged, the digestion, and finally the appetite, entirely fail, and the patient is sustained for some days after by stimulants alone, until furious delirium sets in.

This madness is not due to the stoppage of an accustomed stimulant, for it often sets in while the subject is in the full use of it, but it is plainly due to alcoholic poison and the absence of proper nutritive matter in the blood. I think I may add another cause, which has often something to do in causing the delirium, and certainly much to do in causing death, under some modes of treatment, and that is suppressed excretions. So long as the stomach is intact, and the appetite and digestion are good, an immense quantity of stimulant may be disposed of without serious immediate consequences; but when the organs finally, from constant excitation, become engorged, nutrition ceases, and the alcohol is retained more in the blood, instead of being carried off by excretions, and a wild delirium soon follows.

It is plain, under these circumstances, that the indications are to establish the excretions, disgorge the system of the alcoholic poison, and to introduce proper nutriment. The first two are accomplished by one and the same means. The stomach is generally irritable; at least there is frequent vomiting; but it is owing to the accumulation in the stomach of morbid secretion, rather than from inflammation or even irritation; for calomel in small doses, frequently repeated, arrests it with great certainty. If the subject is governable, and will take medicine willingly, calomel should be given in two or three grain doses every hour, or oftener, if the case is urgent, until fifteen or twenty grains are given; but if medicine has to be given by force, it is best to give a full dose at once; and this is the better, for in the worst cases the stomach is often not nauseated, and the sedative effect of a large dose of calomel calms the nervous excitement, and at the same time produces the appropriate effect upon the excretory organs and mucous coat of the stomach and bowels. It requires some hours for this effect to be produced, and it is improper to give anything to promote its action upon the bowels under ten or twelve hours, and I think even a longer time would be better, if the case is not urgent. Small and frequently repeated doses of saline medicine are the best after the calomel (sulphate of magnesia is best), which promotes the excretions, disgorges the stomach and bowels, and clears the system of its alcoholic poison, to its great relief. An active cathartic may afford some relief, but the system is not so well disgorged by it; more or less serum from the blood is carried off, causing weakness; while, in the other process, by giving time for the action of the calomel, and then promoting it by gentle but continued means, the organs exercise a selection in excreting, and thereby a large amount of effete matter is discharged, and the patient feels the stronger for it, being freed from an incubus that was weighing it down, and producing apparent exhaustion. After this process we should lose no time in introducing nutriment, and for this purpose milk is almost universally applicable; and as the mucous membrane of the stomach seems to be denuded of its epithelium, the addition of lime-water renders it particularly grateful and soothing. Patients in this condition generally loathe animal substances, but milk is almost always grateful to the taste, and is particularly appropriate, for it furnishes the most innocent solid for the bowels, that have been long deprived of their wholesome stimulus. If that a patient could not take milk, well-boiled corn-meal gruel is the next best diet, most likely to be relished; and for something more substantial, strong, well-seasoned broth, frozen, will be the most likely to agree.

In all acute cases, alcoholic stimulants should be withheld, for they act like a poison, and will often bring back the delirium. Should stimulants be thought necessary, (and it is not often really necessary,) the carbonate of ammonia, or the aromatic spirits of ammonia, are preferable; or it may be proper, in some cases, to allow malt liquor. Opium, in all forms, should be prohibited, until the system is relieved thoroughly of its alcohol, and even then I find that it can generally be omitted; and when it can be the patient recovers sooner and better. The patient is not expected to sleep well, but if the blood is renewed by its appropriate nutriment, natural sleep will soon follow.

Occasionally, when, previous to the debauch which immediately caused the mania, a free use of stimulants had been indulged in for some time, we have an exalted state of the nervous system, attended with hallucinations and sleeplessness, which require special attention. Potent stimulants operate badly, and opium alone does not operate well, though in large doses sleep may be forced, though not without some risk, in some cases, to the brain; but equal parts of morphia and tart. antimony, given in small and repeated doses, will soon calm the nervous system and induce sleep, without injury either to the brain or stomach. There is nothing that cools off the heated imagination in these cases like nauseating doses of tart. antimony, and opium in some form may be added, if it is thought necessary. The too general opinion that sleep is the all-important thing in this disease, has led to fatal errors in treatment. Opium, given freely, as it often and very generally is, while the blood is charged with alcohol, produces an unfavorable effect upon the nervous system, and tends to check the excretions, which are already diminished, and the patient, without being narcotised, often goes into a stupid state resembling the effects of uremic poison; and if about one-half (about the usual proportion), by the vigor of their constitutions, weather it in spite of all poisons imposed upon them, they recover slowly, and their organs are left in a bad condition.

I have no record of cases, but within the last ten years a large number have been treated at my Infirmary, by the different house surgeons, under my general directions, and I do not recollect of a single death, unless there was some grave complication more important. In delirium tremens, where it may be proper to keep up the accustomed stimulant to a certain extent, it is generally necessary to attend to the nourishment; for it will be found that the nutritive process is defective and the stimulant will afford but temporary relief, unless this function is restored; or, if it is persisted in without, it will produce the more acute form of mania.

With this view, calomel and other medicine proper to disgorge the stomach and digestive organs, may be proper, and special attention to diet. As in the different forms of other diseases, these two approximate each other in character, and require, in treatment, corresponding modifications. In my early experience in the Charity Hospital, I adopted the opium treatment, and it was not by a sudden change to the opposite extreme, that I arrived at my present mode of treatment, but by degrees, as the true nature of the disease became apparent, and the frequent ill-effects of opium warned me of my error.

In the effect of calomel and salines in this disease, in disgorging the abdominal viscera, and particularly in relieving the stomach, when subject to morbid secretions; and in the effect of tart. antimony and morphia, in subduing morbid sensibility of the nervous system, we discover a therapeutic law that may be usefully applied to other diseases. In engorgements and effusions resulting from obstructed circulation, particularly from the heart, this method is admirably adapted. From the venous engorgement, the functions of digestion and assimilation are almost suspended, the blood becomes impoverished, the skin becomes of a bronze color, and palpitation and other nervous symptoms result in a great measure from it. The dropsical effusion in these cases is generally looked upon as the chief feature of the disease, or consequence of the heart disease, and active hydragogues and diuretics are usually resorted to, when, in reality, if we dis gorgə the organs and establish nutrition, the watery effusion will disappear without trouble. In some cases it takes a large amount of calomel to produce the effect, but it is sure, if persisted in. When I say a large amount, I mean fifty or sixty grains, in small doses, such as will not derange the stomach. When the effect has once been produced, it can easily be kept up, and at the same time the stomach be left free to take the much-needed nutriment. The calomel may be given at night, and early in the morning the appropriate saline or hydragogue, and in two or three hours after the patient will digest better than before, by reason of the improved circulation of the digestive organs. In this way the disgorging process can be kept up, and at the same time the impoverished blood renewed.

In many painful nervous affections, the antimony and morphia are very appropriate, and afford the desired relief with much less injury to the system than by opium or any anodyne alone. In nervous or spasmodic colic, where the patient often suffers excrutiating pain, this combination, if given early, before organic changes take place, not only gives ease very promptly, but relaxes the organs and prepares them for the action of purgatives, if they should be thought necessary. I have given the antimony, combined with Battley's sedative, in low typhoid or nervous fever, when attended with occasional violent neuralgic pains in the head or elsewhere, with the happiest effect, when the sedative, taken alone in a sufficient dose to relieve the pain, produced bad effects. In exalted states of the nervous system and nervous delirium generally, when interference is thought necessary, it is highly useful.—*N. O. Med and Surg. Jour.*

